# Oils and Bioactive Lipids: Quality, Stability, and Functionality

**DOI:** 10.3390/foods10061248

**Published:** 2021-05-31

**Authors:** Diana Ansorena, Iciar Astiasarán

**Affiliations:** 1 Department of Food Science, Nutrition and Physiology, School of Pharmacy and Nutrition, Universidad de Navarra, 31009 Pamplona, Spain; iastiasa@unav.es; 2 Instituto de Investigación Sanitaria de Navarra (IDISNA), 31008 Pamplona, Spain

The dietary consumption of positive bioactive lipids has been shown to be beneficial to human health and to decrease the risk of non-communicable diseases. Omega-3 fatty acids, plant sterols (phytosterols and phytostanols), lipid-soluble vitamins, and antioxidants are among the most studied. These can be naturally present in foods, or added as specific functional ingredients during food processing. In this sense, new sources of these types of compounds are investigated and food reformulation strategies are explored in order to increase the presence of dietary bioactive lipids in foods. However, different challenges have to be faced when incorporating bioactive lipids into beverages and foods, such as susceptibility to oxidation during manufacture and storage, low water solubility, and modification of sensory properties. Consequently, strategies aimed to increase the stability of physical and chemical properties of bioactive lipids are of the utmost interest in order to maintain their functionality and to ensure their bioaccessibility. Moreover, quality, stability, and functionality studies of new oils are needed so that their use in the industry may become a reality.

This Special Issue (SI) is comprised of six articles dealing with relevant topics regarding food lipids.

The first paper (Li et al. [1]) discusses the quality and characterization of oils, by analyzing cucumber, tomato, pumpkin, and carrot seed oils with ultra-performance convergence chromatography (UPC2), combined with quadrupole time-of-flight mass spectrometry (Q-TOF MS). They reported the presence of 36, 42, 39, and 27 different TAGs; a relevant finding, as it was the first time most of them were identified in these oils. Whereas linoleic acid was the most abundant fatty acid in the TG of cucumber, tomato, and pumpkin seed oils, oleic acid was the main finding in carrot seed oil. The authors concluded that the more knowledge achieved about new sources of bioactive lipids, the better selection of appropriate oils with specific functions can be made when developing functional foods in the future.

In this regard, high-quality (HQ) milk was explored as source of vitamin D by Mandrioli et al. [2]. Italian legislation regulates certain parameters in order to allow milk to be qualified as HQ milk. Although some nutritionally key features are needed for this qualification (fat, protein, or lactic acid content), vitamin D is, so far, not included among them. Vitamin D is a fat-soluble vitamin known for its essential role in bone metabolism, regulation of glucose levels, prevention of infections and, if properly supplemented, increased resistance to SARS-CoV-2, as pointed out by the authors of the study. In their work, vitamin D_3_ content was analyzed in HQ milk obtained from Italian cowsheds and supermarkets. Values up to 17.0 µg ± 2.0 µg vitamin D_3_ per liter of milk were found, regardless the amount of fat in the samples. It should be highlighted that the analysis of vitamin D in this matrix is a laborious determination, due to matrix complexity, which requires high sensitivity and specificity methods.

Grajzer et al. [3] analyzed 33 cold-pressed seed oils obtained from several brands of walnut, rosehip, camelina, milk thistle, flax, and pumpkin. They determined fatty acid composition, plant sterols, and tocopherol content in order to provide data for characterizing their lipid fraction. Additionally, total phenol content, antiradical scavenging capacity, and the presence of compounds affecting lipid stability (chlorophylls, Cu, and Fe) were assessed in these oils after 3 months of storage. The authors discussed the relationships, synergisms, and complex interactions among pro and antioxidant molecules that may take place during the shelf life of the analyzed oils. In this sense, the ratio of phytosterols to linolenic acid was suggested as an interesting parameter to evaluate lipid stability.

Whenever food safety is compromised, lipid stability is a major concern. When cholesterol is oxidized (either by heating or by light), cholesterol oxidation products (COPs) are formed, which are widely known for their role in the etiology of atherogenesis and inflammation. Dietary COPs can be absorbed in the gut, therefore minimizing the intake of these molecules might contribute to a better health status. Verardo et al. [4] analyzed two types of egg products (pasteurized and spray-dried eggs), which were further incorporated into biscuit formulations. After baking the biscuits, the samples formulated with pasteurized eggs reported 23.7 µg COPs/g fat content, whereas those containing pasteurized/spray-dried eggs showed 20.4 µg COPs/g fat. However, they differed in the distribution of the individual COPs. Although both samples showed 7-β-HC and 7-KC as the main COPs, samples formulated with spray-dried eggs reported higher amounts of β-epoxide isomers. Moreover, the authors highlighted that spray-drying at lab scale produced lower amounts of COPs compared to the values obtained at industrial scale. This highlighted the importance of controlling food and ingredient processing conditions in order to minimize the presence of potentially toxic compounds in foodstuffs.

Finally, two papers of this SI dealt with the evaluation of the functionality of lipid compounds present in perilla [5] and in dabai pulp oils [6] in human and animal studies. Perilla oil (PO), known for its high content in omega-3 α-linolenic acid (54%–64%), was studied by Hashimoto et al. [5] as a potential supplement that may favor mental health. In this work, the mental condition of healthy Japanese adults was assessed after 12 months of a nutritional intervention with 7 mL of PO or 7 mL olive oil, which served as placebo. The study showed that Self-Rating Depression Scale (SDS) and apathy scores improved significantly in the PO-administered group. Although the mechanism by which the treatment with PO improved mental condition was not well understood, the authors suggested it may be the result of an adequate balance between serotonergic and adrenergic neuronal activity. In addition, PO supplemented volunteers clearly showed higher linolenic and eicosapentaenoic blood levels, whereas no noticeable side effects were reported during the intervention.

In the case of dabai pulp oil (DPO), Kadir et al. [6] evaluated its efficacy against hypercholesterolemia elicited by a high-cholesterol diet in rats, hypothesizing that DPO could be used as a new source of vegetable oil or solid fat. DPO is a new oil extracted from the pulp of *Canarium odontophyllum*, an exotic fruit from Malaysia. Its lipid profile is characterized by near equal percentage of saturated fatty acids (SFA) and monounsaturated fatty acids (MUFA), at about 40% each. Their results showed that supplementation with DPO in hypercholesterolemic rats for 30 days produced a hypocholesterolemic effect (significant reduction in total cholesterol, triglyceride, and 3-hydroxy-3-methylglutaryl-CoA reductase) accompanied by a significant reduction in inflammatory markers (C-reactive protein, interleukin-6, and tumor necrosis factor-α), and lipid peroxidation (MDA). Other biomarkers related to the antioxidant capacity of rats also improved at the end of the intervention. The authors suggested DPO as a plausible healthy alternative fat in the future.

In summary, not only new sources of bioactive lipids are highlighted in this SI, but also the importance of having reliable analytical methods to determine the variety of chemical structures present in these lipids. Moreover, the complex interaction between protective and pro-oxidant factors affecting food lipid stability is shown, as well as the potential health benefits of lipid compounds. Food lipids and their nutritional and health implications remain a popular topic for food scientists, and it seems likely to be a challenging field for the near future.
